# Pathway Distiller - multisource biological pathway consolidation

**DOI:** 10.1186/1471-2164-13-S6-S18

**Published:** 2012-10-26

**Authors:** Mark S Doderer, Zachry Anguiano, Uthra Suresh, Ravi Dashnamoorthy, Alexander JR Bishop, Yidong Chen

**Affiliations:** 1 Greehey Children's Cancer Research Institute, The University of Texas Health Science Center at San Antonio, San Antonio, TX, USA; 2 Department of Medicine, Division of Hematology Oncology, University of Massachusetts Medical School, Worcester, MA, USA; 3 Department of Cellular and Structural Biology, The University of Texas Health Science Center at San Antonio, San Antonio, TX, USA; 4 Department of Epidemiology and Biostatistics, The University of Texas Health Science Center at San Antonio, San Antonio, TX, USA; 5 Cancer Therapy & Research Center, The University of Texas Health Science Center at San Antonio, San Antonio, TX, USA

## Abstract

**Background:**

One method to understand and evaluate an experiment that produces a large set of genes, such as a gene expression microarray analysis, is to identify overrepresentation or enrichment for biological pathways. Because pathways are able to functionally describe the set of genes, much effort has been made to collect curated biological pathways into publicly accessible databases. When combining disparate databases, highly related or redundant pathways exist, making their consolidation into pathway concepts essential. This will facilitate unbiased, comprehensive yet streamlined analysis of experiments that result in large gene sets.

**Methods:**

After gene set enrichment finds representative pathways for large gene sets, pathways are consolidated into representative pathway concepts. Three complementary, but different methods of pathway consolidation are explored. Enrichment Consolidation combines the set of the pathways enriched for the signature gene list through iterative combining of enriched pathways with other pathways with similar signature gene sets; Weighted Consolidation utilizes a Protein-Protein Interaction network based gene-weighting approach that finds clusters of both enriched and non-enriched pathways limited to the experiments' resultant gene list; and finally the *de novo *Consolidation method uses several measurements of pathway similarity, that finds static pathway clusters independent of any given experiment.

**Results:**

We demonstrate that the three consolidation methods provide unified yet different functional insights of a resultant gene set derived from a genome-wide profiling experiment. Results from the methods are presented, demonstrating their applications in biological studies and comparing with a pathway web-based framework that also combines several pathway databases. Additionally a web-based consolidation framework that encompasses all three methods discussed in this paper, Pathway Distiller (http://cbbiweb.uthscsa.edu/PathwayDistiller), is established to allow researchers access to the methods and example microarray data described in this manuscript, and the ability to analyze their own gene list by using our unique consolidation methods.

**Conclusions:**

By combining several pathway systems, implementing different, but complementary pathway consolidation methods, and providing a user-friendly web-accessible tool, we have enabled users the ability to extract functional explanations of their genome wide experiments.

## Background

There exist several public data sources such as Biocarta [1], KEGG [2], WikiPathways [3], Pathway Commons [4], NCBI's Biosystems [5], NCI Nature [6], Reactome [7] and HumanCyc(a member of the BioCyc database) [8] for pathway annotations including cellular process, metabolic process, molecular function, and physiological process. These data sources also provide a variety of information ranging from simple formats, for example a list of genes involved in a specific pathway, to complex information, like the directed graph of biological entities and their effect on each other. There also exist private data sources like Ingenuity [9], Pathway Studios [10], and Protein Lounge (http://www.proteinlounge.com) however they are not freely available.

Pathway information can offer insights for a variety of research including genome-wide gene expression analysis. Gene expression levels detected by microarrays and Next Generation Sequencing (NGS) allow the profiling of gene products that are differentiated between diverse conditions. Likewise, genomic copy number alteration, differential methylation, and other genome-wide profiling experiments result in a list of resultant genes with the capacity to differentiate phenotypic or treatment conditions. Often biological *concepts *are used to describe gene lists [11,12]. The concepts are unifying characteristics that are statistically enriched for the gene list and provide functional insight related to the gene list. Any concept that has a predefined list of genes matching some or all of the experiment's resultant genes is considered enriched and the level of enrichment is statistically quantifiable (relative to random selection). Selection of pathways (concepts) based on the statistical significant enrichment score (ES) is one natural way to infer function from gene expression patterns. Gene Set Enrichment Analysis (GSEA) [13] introduced a Kolmogorov-Smirnov like method that finds enriched pathways by statistical analysis of genes that can be ordered by measurement such as expression fold change. However when no ordering measurement is available, some other means, like Fisher's Exact test is necessary to find enriched pathways.

The development of many genome-wide profiling technologies and the number of pathway data sources has lead to an explosion in the number of pathways to be studied from a single gene set. Chowbina *et al. *[14] discuss the integration of multiple data sources to determine a single collection of pathways that provides functional insight for experimental gene sets. Additionally, they provide an online database (HPD) to give users access to their integrated pathway database containing 999 human pathways. Yu *et al. *[15] have combined several pathway database to create another integrated pathway database (hiPathDB) with 1661 human pathways. To create a similar database, we downloaded pathways from BioCarta, Pathway Commons, NCBI BioSystems, and WikiPathways, and after removing pathways with no gene members, 2,462 pathways remain (as of February 2012) from specific sources including WikiPathways, Reactome, KEGG, NCI Nature, BioCarta and HumanCyc. Our database contains all of the pathway sources of HPD and hiPathDB except Protein Lounge which is not publically available. While larger collection of pathways may appear to be better starting point, the interdependency and/or redundancy within and between databases will skew the statistical assessment of a large gene set and lead to incorrect functional associations. A better solution would be to consolidate the complete list of pathways as non-redundant and representative pathways, enabling better understanding of any experiment including gene set enrichment results.

A natural way to combine lists is by parsing the pathway names, for example, KEGG, Reactome and WikiPathways all contain "Apoptosis". However in this instance although the pathway names are the same, the gene sets are different. Name matching by itself also misses exact gene set matches. "Synthesis and Degradation of Ketone Bodies" from WikiPathways and "Ketone body metabolism" from Pathway Commons contain exactly the same 5 genes. This match would be missed if simple name matching were employed.

Another natural method of pathway consolidation finds some overlap between the gene members of pathways pairs, including exact gene set matches. By observing the same gene set in two pathways they could be consolidated into a single representative pathway. For example, three separate Reactome pathways contain the exact same gene members, "SMAC binds to IAPs", "SMAC-mediated apoptotic response", and "SMAC-mediated dissociation of IAP:caspase complexes". Like the previous example, all three pathways could be consolidated into one "pathway concept" simply by observing the identical gene membership in sets. Among the 2,462 pathways, 312 have exactly the same gene members with at least one other pathway, forming 136 distinct groups. This method would reduce the number of pathways somewhat (2,462 - 312 + 136 = 2,286). To consolidate further, pathways that are similar could be combined when they have sufficiently similar gene sets, however the difficulty is determination of "sufficiently similar".

A naïve way of determining "sufficient similarity" is by using subset relationship. For example, "Regulation of RhebGTPase activity by AMPK" from Reactome contains 10 genes that are all present in the "Insulin Signaling" pathway from KEGG with 138 genes. This situation is typical when aggregating data sources that contain general pathways and pathways that represent specific sub-pathways of the general pathways. Of the 2,462 pathways, 1,681 of them are exact subsets of at least one other pathway. While this might seem to offer a way to significantly consolidate pathways, there exist overlapping pathway gene sets that make consolidation difficult. For example, "Endothelins" from NCI Nature and "TRAIL signaling pathway" of NCI Nature contain all of the 22 genes of the "IL6 pathway" from BioCarta, but "Endothelins" and "TRAIL signaling pathway" are not subsets of each other, making it impossible to combine them by grouping subsets with supersets. Therefore "IL6 pathway" cannot be labelled as a subset of a single pathway, in this case only as a subset of two pathways.

In addition to membership comparison, several methods have been proposed to accomplish pathway clustering by pathway activity similarity (expression, disease phenotypes, etc). Mamitsuka *et al. *[16] proposed a method based on Markov mixture models that relies on microarray expression and the graph of a metabolic pathway. Li *et al. *[17] proposed a method that clusters sub-pathways according to diseases. Because of the diversity of representations of pathways among the publically available databases, we relied on the most simple representation of pathways, their gene members. Similarly, it is possible to find microarray expression values for the gene members, like Fang *et al. *[18], however we chose to keep our gene sets unbiased for specific microarray studies. While this information might be advantageous in specific applications, for many systems biology studies, a generic approach is desired.

**Figure 1:**
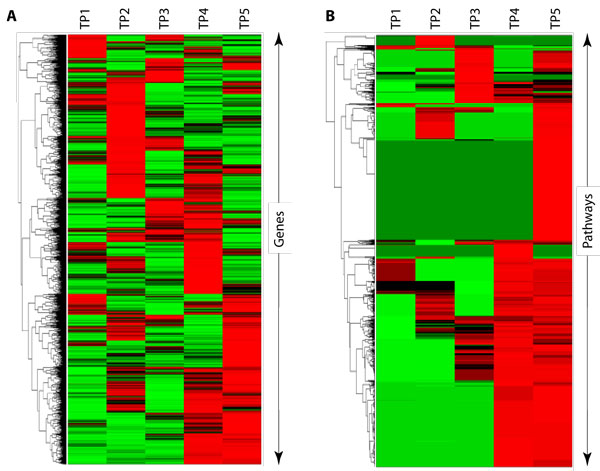
**Hierarchical clustering of expression data at gene level and pathway level**. Hierarchical clustering of (A) absolute gene expression over 5 time points; and (B) pathway expression. Gene expression of samples at 5 different time points (TP1 to TP5) are marked above the heatmap, log2-transformed and normalized gene expression data of each gene were plotted in Figure 1A. Average of absolute gene expression over each pathway were evaluated and then plotted in Figure 1B to reflect the activities of biological pathways.

One online integrate pathway repository, HPD [14], computes a similarity score for all pathway pairs based on overlapping gene sets. A user then can visualize the scores in an "Association Table". Through visualization a user would be able to see similar pathways. Another repository, hiPathDB, only lists all of the pathways that contain at least one of the input genes with no means for determining pathway overlap. We propose three different methods to consolidate multiple pathways into one *pathway concept*. Each provides a slightly different analysis of the concepts describing a single experiment. Figure 1 illustrates how different methods of analysis can draw attention to different aspects of the same data. Hierarchical clustering by Gene Expression (Figure 1A) shows how the absolute expression values for each gene changes over the time course data. Alternatively, clustering by Pathway Expression (Figure 1B) highlights pathway activity over the time course data. Taken together, they provide complementary, but different views of the same data. Our consolidation methods also provide complementary, but different views of the pathway concepts that represent an experiment's resultant gene set. One is tightly associated with the results from gene expression experiments consolidating only enriched pathways. Another uses pathway pair scoring, comparing not only the overlap of the resultant genes, but also weighting by shared interactions between the pathways. This method consolidates both enriched and non-enriched pathways. The third method, independent of any given resultant gene set, combines pathways with other pathways using their gene member and the gene member annotations like ontology or interacting pairs. The first and second pathway consolidation methods combine gene set membership and gene expression analysis to create a consolidated list of pathways specific to the condition studied in the analysis. Therefore, it is tailored to a specific experiment. The third measures the overlap of two sets by a variety of criteria and then groups pathways with the greatest similarity. This creates a set of grouped pathways independent of any experiment that is used for gene set enrichment.

## Methods

### Data collection

All human pathway data presented here were downloaded from four primary data repositories, BioCarta, WikiPathways, NCBI BioSystems and Pathway Commons representing six data sets (update to February 2012). BioCarta (BioCarta), KEGG (obtained via NCBI BioSystems), NCI Nature (obtained via Pathway Commons) and HumanCyc (obtained via Pathway Commons) were contained in one of the data sources as indicated, however Reactome (covered by both NCBI BioSystems and Pathway Commons) and WikiPathways (covered by both WikiPathways and NCBI BioSystems) were contained in two different repositories. For those situations where the names matched between sources the gene identifiers were combined. 2,665 pathways were determined from BioCarta, KEGG, NCI Nature, WikiPathways, Reactome and HumanCyc. 203 pathways with no gene members were removed leaving 2,462 pathways with at least one gene member.

Annotations including Gene Ontology (GO) [19] and protein interactions [20] were downloaded along with GO Slim annotations for the GO entries.

Two gene expression data sets were obtained. The first [21] was used to test our consolidation methods and is provided as an example in the Pathway Distiller framework. Gene expression profiles by Affymetrix HG-U95Av2 were measured at five different time points after treatment with cisplatin in the human ovarian cancer cell A2780. After normalization and filtering steps, thresholding of log-fold-change at 1.0 was performed to generate 5 differentially expressed gene sets that were further combined to form a single resultant gene set (total of 526 genes) that represents this experiment over any time point. The background gene set contained all of the probes of Affymetrix HG-U95Av2, regardless of expression level that map to NCBI Entrez GeneIDs (total of 8000 genes).

### Pathway enrichment and consolidation

One-sided Fisher's Exact test finds *p*-values for all pathways that contains at least 1 of the user supplied resultant gene set compared to the human genome or a user supplied background gene set. Three methods were developed to focus on different but complementary cluster types for the resulting pathways. One method focuses only on enriched pathways unique to the experiment's resultant gene set. Another method clusters both enriched and non-enriched pathways using scores derived from interactions within each pathway involving the resultant genes. The final method independent of the resultant gene set finds cluster of pathways based on gene membership, GO ontology and protein-protein interactions. This is the only method that can be pre-determined, independent of experiments' resultant gene lists.

#### A. Consolidation of enriched pathways

Given the initial resultant gene set and the initial most enriched pathway, we iteratively reduce the list of enriched pathways and the resultant gene set by removing the genes in the most enriched pathway from the resultant gene set and recomputing enrichment *p*-values for the remaining pathways using the reduced gene set. Any pathway that is no longer enriched is consolidated with the most enriched pathway for this iteration. Because each iteration starts with removal of shared genes based on enrichment *p*-values, the order of gene removal is different for each resultant gene set and therefore specific to that experiment. We perform the consolidation procedure as follows,

Pathway Consolidation Procedure 1: *Enrichment Consolidation*

1) Start with a set of all enriched pathways (each pathway is defined as a collection of genes, or *s_i _*= {*g_i_*}), or *S*^0 ^= {*s_i_*: *p*_*i*_Fisher_(*D*) ≤ 0.05}, where *D *is the differential expressed gene set or resultant gene set, *p*_*i*_Fisher _is the enrichment *p*-value of pathway *s_i _*in gene set *D*; a set of genes supporting all pathways, or *G*^0 ^= {*g_k_*: *g_k _*∈ any *s_i_*}, and initialized the selected pathways set *P*^0 ^= ∅;

2) Find the most enriched pathway *s_j _*where *s_j _*∈ *S*^0 ^*& p_j _*≤ *p_i_*, for all *i *≠ *j*;

3) Upon obtaining *s_j_*, we update *S*^1 ^= {*S*^0^\*s_j_*}, *G*^1 ^= {*G*^0^\*g_k_*; *g_k _*∈ *s_j_*}, and selected pathway set *P*^1 ^= *P*^0 ^∪ {*s_j_*};

4) Update pathway enrichment *p*-values, since we removed genes contained in *s_j _*from all other pathways;

5) Update remaining pathway set by removing non-significant pathways after removing genes contained in previous enriched pathway, *S*^1 ^= {*S*^1^\*s_i_*: *p*_*i*_Fisher_(*D*) > 0.05}; and

6) Repeat steps 2-5, stop when *S^m ^*= ∅.

By starting with the most enriched pathway each iteration, each pathway concept is represented by the most enriched pathways for the resultant gene set.

#### B. Weighted consolidation with resultant genes

Weighted Consolidation first orders pathway pairs by scores that incorporate information about the number of overlapping resultant genes and the interactions among those resultant genes. Second, it adds each pair from highest to lowest score into a cluster. For weighting we followed the method outlined by Fang *et al. *[18] to determine weight (*W_i_*) for each gene (*g_i_*) within each of the pathways (*s_j_*) by comparing the number of known and predicted interactions [20] between *g_i _*and the other genes in *s_j_*, against the number of known and predicted interactions between *g_i _*and the other genes in the genome. Suppose that *g_i _*has no specific functional association with genes in pathway *s_j_*; then the number of interactions *X_i _*of *g_i _*to others in a given pathway is expected to follow a hypergeometric distribution,

(1) $$P\left(X_{i}=x|N,M_{i},K_{j}\right)=\frac{\left(\begin{array}{c} M_{i}\\ x \end{array}\right)\left(\begin{array}{c} N-M_{i}\\ K_{j}-x \end{array}\right)}{\left(\begin{array}{c} N\\ K_{j} \end{array}\right)}$$

where *M_i _*is the number of interactions of *g_i _*to others in the genome, *N *is the number genes in the genome, and *K_j _*is the number of genes in pathway *s_j _*with the expected number of interactions *E*(*X_i_*) of gene *i *derived as

(2) $$E\left(X_{i}\right)=\frac{M_{i}K_{j}}{N}$$

for gene *g_i_*, *i *= 1, 2, ..., *K_j_*. The observed number of interactions between genes in *s_j _*is likely to be significantly larger than *E*(*X_i_*), when there is a specific function association of *g_i _*in pathway *s_j_*, thus a larger weight. In the algorithm, they rescaled this weight to quantify the relative association strength *W_i _*of *g_i _*as,

(3) $$w_{i}=X_{i}-E\left(X_{i}\right),$$

(4) $$W_{i}=\left\{\begin{array}{cc} log_{2}\left(w_{i}+2\right), & \;w_{i}>0\\ 1, & \;w_{i}\leq 0 \end{array}\right)$$

Although Fang *et al. *included co-expression and functional annotation associations between genes, we limited the association count to the interactions found in one of our earlier implementations of the protein interaction database, InterologFinder [8,20]. Gene expression could be determined for the gene members, however the experiments themselves are tissue and condition specific. We chose to leave Pathway Distiller unbiased for any one experiment by not incorporating gene expression information.

The pathway consolidation procedure is implemented by utilizing a similarity score confined to the resultant gene set, *D*, with weights generated from the number of interactions within a given pathway. Upon obtaining a similarity score (Eq. 5) for each pathway pair, we iteratively process the pathway pairs from high to low scores either adding them to existing clusters or forming a new cluster with the pair. Specifically, we perform the consolidation procedure as follows,

Pathway Consolidation Procedure 2: *Weighted Consolidation*

1) Start with a set of all pathways (each pathway is defined as a collection of genes, or *s_i _*= {*g_i_*}, each gene within *s_i _*has a unique weight *w_i _*according to its relative association with the other genes in pathway *s_i _*as described in Eqs. (1-4); a resultant gene set, *D*, is also required;

2) Determine a similarity score for each pathway pair where the weights for overlapping resultant genes between the pathways are compared against the weights between all resultant genes in the pathway pair,

(5) $$Similarity\left(s_{i},s_{j}\right)=\frac{\sum_{g_{k}\in \left(s_{i}\cap s_{j}\cap D\right)}w_{k}}{\sum_{g_{k}\in \left(\left(s_{i}\cup s_{j}\right)\cap D\right)}w_{k}}$$

3) For each unique pathway pair, ordered by their similarity score, add one, both or neither pathway to a cluster *c_k _*that is a member of all clusters *C *= {*c_k_*} and made up of a set of pathways.

a. If neither *s_i _*nor *s_j _*already belong to a cluster in *C*, create a new cluster *c' *= {*s_i_*, *s_j_*}, add cluster *c' *to *C*;

b. If *s_i _*is an element of *c_k_*, but *s_j _*is not a member of any cluster in *C*, add *s_j _*to *c_k_*, similarly handled vice versa;

c. If both *s_i _*and *s_j _*are members of any cluster in *C*, do nothing.

4) Repeat Step 3) for all pathway pairs.

#### C. *De novo *pathway clustering

**Figure 2:**
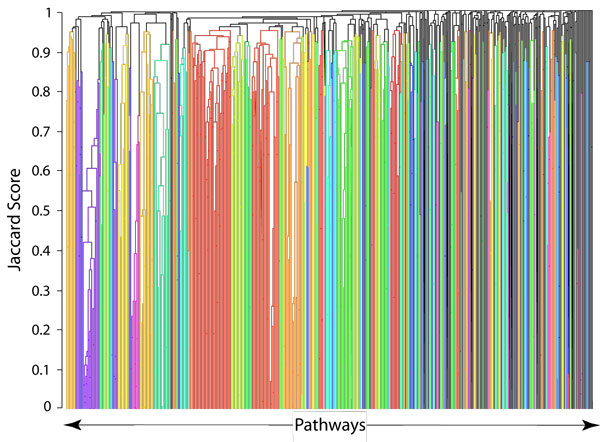
**Hierarchical clusters of aggregated pathways**. Dendrogram generated by using PPI similarity measurement and 250 clusters were colored automatically to illustrated grouping effect.

To cluster pathways without experimental information, a hierarchical clustering-based method was developed in MATLAB using the Statistics Toolbox (Mathworks, Natick, MA). After finding similarity measurements between pathways, the linkage algorithm is used to link pairs of pathways to form a hierarchical cluster tree. The Cluster function was used to either choose a particular cut-off threshold for groupings, or to choose how many clusters the user desires. As shown in Figure 2, the clustering is effectively a horizontal cut of the clustering dendrogram. The number of clusters is relative to the minimum Jaccard coefficient [22] threshold required to combine clusters when each pathway starts in its own cluster. By choosing different cluster count thresholds, the user can have different similarity requirements. The lower the cut-off threshold the fewer the number of clusters and the more pathways grouped in each cluster. To find an actual measure of pathway similarity, the Jaccard coefficient and distance are used as shown in (6) and (7).

(6) $$J\left(s_{i},s_{j}\right)=\frac{\left|A_{i}\cap A_{j}\right|}{\left|A_{i}\cup A_{j}\right|}$$

(7) $$d_{i,j}=1-J\left(s_{i},s_{j}\right)$$

where |·| is the cardinality of a set, and *s_i _*and *s_j _*are *i*th and *j*th pathways similarly defined earlier, and *A_i _*and *A_j _*are attributes of genes in pathways *s_i _*and *s_j_*. A total of three different gene attributes were looked at as the common term: 1) gene membership, 2) "Guilt-by-function" via Gene Ontology, and 3) "Guilt-by-association" via protein-protein interactants. Our algorithm handles all in the same manner by finding the Jaccard distance between two pathways based on set commonality. This forms a Jaccard matrix, that represents the similarities between all pathways.

For pathway consolidation based on Gene Ontology and protein-protein interactions, a map was created to link pathways to GoSlim terms, and to protein-protein interactants creating new member sets from the original sets used in (6) and (7). Or

(8) $$A_{i}\left(s_{j}\right)\leftarrow \left\{\begin{array}{c} \left\{g_{i};g_{i}\in s_{j}\right\}\\ {⋃}_{g_{i}\in s_{j}}\text{GOSlim}\left(g_{i}\right)\\ {⋃}_{g_{i}\in s_{j}}\text{PPI}\left(g_{i}\right) \end{array}\right)$$

The mapping in (8) takes each gene in the pathway, or creates a new set including the GoSlim ancestors of the original gene's GO terms, GOSlim(*s_i_*); or creates a new set including any gene that is known to interact with the genes known to be in the pathway PPI(*s_i_*). The exact procedure is follows,

Pathway Consolidation Procedure 3: *de novo *Consolidation

1) Start with a set of all pathways (each pathway is defined as a collection of genes), or *s_i _*= {*g_i_*}.

2) Determine the method of similarity measure (membership, guilt-by-function, or guilt-by-association, Eq. 8), and then evaluation Jaccard coefficient by Eqs. 6-7;

3) Perform hierarchical clustering to obtain desired specificity of pathway clusters defined by the user. User indicates Jaccard Score cut-off, which defines the clustering stop point.

### Pathway distiller framework

We developed a web-based application, the Pathway Distiller (http://cbbiweb.uthscsa.edu/PathwayDistiller), which provides access to our integrated pathway database base, pathway enrichment and three methods of pathway consolidation. It builds on the Sidekick [23] framework. Pathway Distiller was built using Adobe Flex and Action Script 3 which produces a Adobe Flash movie that runs in a user's web browser. The back-end automatic data download, processing and query availability was created using the SideCache [24] framework that is implemented in Java and designed to run as a Java Servlet in a container such as Apache Tomcat [25]. Users can upload a set of genes (resultant gene set) to access all components of the Pathway Distiller functions or use a supplied sample gene set. The *de novo *pathway clusters are also available for download.

## Results

**Figure 3:**
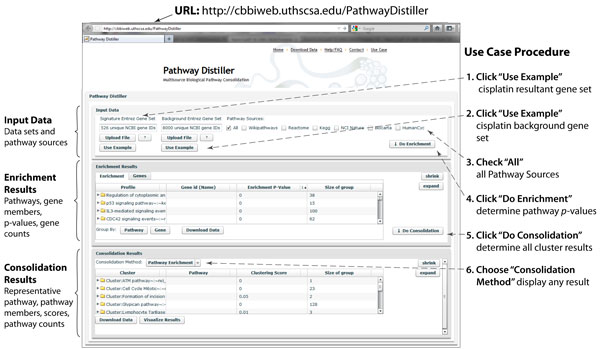
**Pathway distiller screen shot of sample analysis**. Steps for a typical resultant gene list analysis and pathway cluster discovery. Step 1: click to use sample resultant gene set. Step 2: click to use sample background set. Step 3: check all pathways. Step 4: click to do one-sided Fisher's Exact pathway enrichment; fill Enrichment Results grid. Step 5: click to do pathway consolidation; fills Consolidation Results grid; shows default consolidation method. Step 6: click to select from consolidation methods.

Using the Pathway Distiller framework as outlined in Figure 3, we describe the process of taking a resultant gene list (e.g., derived from a differential expression analysis method) and finding enriched pathway concepts (a set of pathways derived from a consolidation procedure) that provide function insights for the genomic data. Further, we outline how the various consolidation methods find different descriptions of the resulting pathway concepts related to the supplied resultant gene list.

### Cisplatin case study and framework use case

The web-based application, Pathway Distiller, as shown in Figure 3 and accessible at http://cbbiweb.uthscsa.edu/PathwayDistiller, facilitates resultant gene set analysis using pathway enrichment and consolidation. Using the supplied cisplatin resultant gene set as a Use Case, we import (1. Click) the 526 differential expressed genes induced by cisplatin treatment (see Methods) and the 8000 background genes (2. Click). The user is able to upload their own resultant gene set and background gene set using the Upload File button. The background gene set could be all of the probes for a given microarray platform, while the resultant gene set would include only the differentially expressed genes. By default (or when background gene set is not presented), the entire gene set of human genome will be used as the background. In Step 3 (3. Check) we leave the default option (All) to find the enrichment score for each of the 2,462 pathways maintained by our backend data management module. The user is able to limit the pathway sources. Step 4 (4. Click) fills the Enrichment Results grid with the pathways with at least one of the resultant genes. For the cisplatin case study, this results in 1456 pathways. Step 5 (5. Click) fills the Consolidation Results grid. The default method, Pathway Enrichment (EC), provides the 12 pathway concepts mostly closely aligned with the Enrichment Results. Step 6 (6. Choose) displays the results of the other consolidation methods. Additional features of Pathway Distiller include flexible viewing options and links to external data sources for most of the pathways and genes. A more thorough use case is available on the Pathway Distiller website including screen shots explaining Pathway Distiller's functionality.

In our cisplatin case study, by sorting the grid by enrichment score, 177 pathways were found to be significantly enriched with *p*-value ≤ 0.05 (one-sided Fisher's Exact test). After the pathways are consolidated, the user is able to choose from all of the consolidation methods using the Consolidation Method drop down box. This displays the pathway concepts (clusters), their member pathways, the clustering score and the number of pathways within the pathway concept. Each pathway concept, is labelled with the most significant pathway in the group. The clustering score for each method is the lowest enrichment score of the individual pathways within the cluster.

For our cisplatin example, the Consolidation Results grid first displays the EC consolidation results providing insight into the similarity of the significantly enriched pathways highly according to the resultant gene list's pathway enrichment. By choosing the Weighted Consolidation (WC) option, the user finds pathway concepts related to the specific input resultant gene set, however it includes all of the 1,456 pathways with at least one cisplatin-induced differentially expressed gene. Of the 12 pathway concepts for the EC method, 1 of them contains 128 pathways and 4 of them contain a single pathway. The WC method finds a maximum of 29 and minimum of 2 pathways in the 318 pathway concepts.

When more specific functional insights are required, by choosing from the 3 *de novo *Consolidation methods (DC) a user is able to have more fine-tuned control of both the means to measure pathway similarity and the number of concepts to determine. The DC naming convention Pathway Distiller uses is as follows, the first half of each option in Step 6 indicates the gene attribute from which the Jaccard score is derived. Specifically, membership will provide gene membership, GoSlim "Guilt-by-function" via Gene Ontology, and PPI "Guilt-by-association" via gene interactions. Each attribute is further subdivided into 8 cluster count thresholds indicated in the second half of the DC naming convention. Cut-offs of 5, 10, 20, 40, 50, 100, 200 and 500 provide the user fined-tuned control of the number of expected clusters and the resulting size of each cluster. Together with EC and WC, the DC results for the cisplatin-induced differentially expressed genes are described in the first two columns of Table 1. In Step 6, choosing GOSlim10 (maximum of 10 concepts weighted by functional similarity) displays the 2 pathway concepts with at least one of the 1456 pathways (Table 1 column 1). Of these two concepts only one of them contains a significantly enriched pathway (Table 1 column 2). membership10 and PPI10 each only produce 1 concept that overlaps the pathways with at least 1 cisplatin resultant gene. In Step 6, GOSlim100 (maximum of 100 concepts) displays the 51 pathway concepts with at least one of the 1456 pathways and by sorting 8 of them contain at least one enriched pathway. Summarized in the Table, membership100 and PPI100 find larger number of concepts than their corresponding 10 cut-offs (42 and 16 respectively) and of these even fewer contain enriched pathways (10 and 2 respectively). The final DC type/cut-off combination of 500 for GOSlim, membership and PPI finds 215, 263, and 194 concepts with 21, 46, and 24 concepts with at least one enriched pathway for each, respectively.

**Table 1:** Pathway cluster counts for cisplatin resultant gene set

Method	Number of Pathway Concept	Number of Concepts with Enriched Pathways	Min number of pathways in a concept	Max number of pathways in a concept
Enrichment Consolidation	12	12	1	128
Weighted Consolidation	318	74	2	29
GOSlim10, membership10, PPI10	2, 1, 3	1, 1, 1	1, n/a, 1	1456, 1456, 1453
GOSlim100, membership100, PPI100	51, 42, 16	8, 10, 2	1, 1, 1	1116, 793, 1248
GOSlim500, membership500, PPI500	215, 263, 194	21, 46, 24	1, 1, 1	727, 79, 560

Note: n/a - indicates all pathways formed a single cluster

As expected in the fine-tuning of the DC methods the maximal number of pathways in pathway concepts decreases as the number of pathways increases. For example all of the 1456 pathways with at least one cisplatin induced differentially expressed gene are clustered together for GOSlim10, however the maximal number of pathways in a pathway concept for GOSlim500 is 727. In Table 1, we additionally provide results for allowing 100 and 500 clusters to demonstrate the incremental gain of number of pathway concepts, thus provide precise description of the input gene set.

The correct cut-off level is dependent on the user's needs both in terms acceptable size and number of clusters and interest in specific pathways. For example, if a user is interested in a specific group of pathways using a lower cut-off like 40 combines them into the same cluster with very few other pathways, this cut-off would be appropriate. In membership40, the Sphingolipid metabolism cluster contains only 6 pathways which is very manageable. However, at the same cut-off, the Nucleotide Metabolism cluster contains 170 which might be too large to inspect. Using membership100 reduces the Nucleotide Metabolism cluster to 19 pathways.

We investigated whether the pathway concepts obtained from the EC and WC algorithms are significant when compared to random draws of the same number of genes from the genome. We assume that genes contained in these pathways concepts show much stronger interaction, comparing to genes randomly selected from the genome. Table 2 shows the *p*-values of the number interactions of genes within pathways concepts when comparing to the same number of genes drawn at random 1000 times. Other than 3 pathway concepts (Methionine Degradation I, Nucleotide Metabolism, and Vitamin C in the Brain pathways), genes in most pathway concepts show significantly strong interaction.

**Table 2:** Cluster estimation of randomness results

Pathway	Enriched Consolidation *p*-value*	Weighted Consolidation *p*-value*
Androgen Receptor Signaling Pathway	< 10^-150^	< 10^-150^
ATM Signaling Pathway	< 10^-150^	< 10^-150^
Cell Cycle, Mitotic	< 10^-150^	< 10^-150^
Formation of Incision Complex in GG-NER	1.81 × 10^-11^	2.11 × 10^-13^
Glypican Pathway	< 10^-150^	< 10^-150^
Lymphocyte TarBase	3.51 × 10^-19^	< 10^-150^
Methionine Degradation I (tohomocysteine)	2.40 × 10^-4^	0.14
mRNA Processing	1.96 × 10^-67^	5.81 × 10^-128^
Myometrial Relaxation and Contraction Pathways	6.88 × 10^-45^	2.64 × 10^-63^
Nucleotide Metabolism	0.11	4.05 × 10^-17^
p63Transcription Factor Network	3.55 × 10^-31^	8.67 × 10^-72^
Spliceosome Prp19/CDC5L complex	6.13 × 10^-4^	3.20 × 10^-9^
Vitamin C in the Brain Pathway	0.36	3.39 × 10^-14^

Note: *p*-value is derived from χ^2^Goodness of Fit

### **Overview of **Table 3-6 **organization**

Tables 3, 4, 5, 6 show results from a variety of resultant gene sets. The tables are organized with rows of pathways and in each column how the pathways are grouped in that consolidation method. The columns are divided to facilitate reading the tables in column-wise directions. In other words, the divisions of a column show how one clustering methods groups the pathway rows. Each division represents a pathway concept. Pathways that do not consolidate into pathway concepts are not contained in their own division and do not share similar symbols.

**Table 3:** P53 overlapping cluster membership

Pathway Name, (enrichment *p*-value)	*de novo *Gene Membership	*de novo *Gene Ontology	*de novo *Protein Interactants	Weighted Consolidation	Enrichment Consolidation
Direct p53 effectors, (*p *= 1.16 × 10^-9^)	■	■	■	■	■
p53 pathway, (*p *= 6.19 × 10^-9^)	■	■	■	■	■
					
p53 signaling pathway, (*p *= 9.84 × 10^-7^)	▲	■	■	▲	■
			
p53-dependent G_1 _DNAdamage response, (*p *= 0.15)	●	●	■	▼	n/a
p53-dependent G_1_/S DNA damage checkpoint, (*p *= 0.15)	●	●	■	▼	n/a
p53-independent DNA damage response, (*p *= 0.25)	●	●	■	●	n/a
p53-independent G_1_/S DNA damage checkpoint, (*p *= 0.25)	●	●	■	●	n/a

Note: matching symbols in a column indicate shared cluster membership. n/a indicates non-enriched pathway not included in any cluster. Dividers and matching symbols indicate pathways groups within a consolidation method.

**Table 4:** RNAi resultant gene set overlapping cluster membership

Pathway Name, (enrichment *p*-value)	*de novo *Gene Membership	*de novo *Gene Ontology	*de novo *Protein Interactants	Weighted Consolidation	Enrichment Consolidation
Base excision repair (*p *= 0.58)	■	●	■	■	n/a
Mismatch Repair (*p *= 0.75)	■	●	■	■	n/a
					
Homologous Recombination Repair (*p *= 0.73)	■	■	■	■	n/a
DNA damage response (*p *= 0.09)	■	■	■	■	n/a
					
Nucleotide Excision Repair (*p *= 1.99 × 10^-4^)	■	■	■	▲	■
Proteasome (*p *= 3.4 × 10^-3^)	■	■	■	►	▲
Basal Transcription (*p *= 2.97 × 10^-11^)	■	■	■	◄	►
Ribosome (*p *= 6.65 × 10^-3^)	■	■	■	♦	▼
					
TOR pathway (*p *= 2.90 × 10^-3^)	▲	■	■	▼	●
Notch Signaling Pathway (*p *= 3.05 × 10^-4^)	▲	■	■	◘	◄
			
Glutathione Biosynthesis (*p *= 0.11)	►	►	►	●	n/a

Note: RecQ helicases and ATPase were not found among the human pathways. *p*-values are derived from one-tail Fisher's Exact test, before multiple test correction. Dividers and matching symbols indicate pathways groups within a consolidation method.

**Table 5:** HPD gene set overlapping cluster membership

Pathway Name, (enrichment *p*-value)	HPD similarity grouping	*de novo *Gene Membership	*de novo *Gene Ontology	*de novo *Protein Interactants	Weighted Consolidation	Enrichment Consolidation
Aurora B signaling (*p *= 1.32 × 10^-2^)	■	▲	■	▲	▲	■
					
Aurora A signaling (*p *= 1.39 × 10^-4^)	■	■	■	■	■	■
Signaling by Aurora kinases (*p *= 3.27 × 10^-4^)	■	■	■	■	■	■
				
FOXA transcription factor networks (*p *= 2.23 × 10^-4^)	●	●	■	●	●	■
FOXA1 transcription factor network (*p *= 6.53 × 10^-5^)	●	●	■	●	●	■
					
FOXA2 and FOXA3 transcription factor networks (*p *= 1.38 × 10^-2^)	●	●	■	♦	♦	■
		
Fanconi Anemia pathway (*p *= 8.36 × 10^-3^)	▲	▼	►	►	►	■
						
ATRBRCA_PATHWAY (*p *= 6.75 × 10^-3^)	▲	►	►	►	►	■
						
ATM mediated phosphorylation of repair proteins (*p *= 1.93 × 10^-3^)	►	►	►	▼	►	■
Recruitment of repair and signaling proteins to double-strand breaks (*p *= 2.58 × 10^-3^)	►	►	►	▼	►	■

Note: *p*-values are derived from one-tail Fisher's Exact test, before multiple test correction. Dividers and matching symbols indicate pathways groups within a consolidation method.

**Table 6:** TP53 overlapping cluster membership

Pathway Name, (enrichment *p*-value)	HPD similarity grouping	*de novo *Gene Membership	*de novo *Gene Ontology	*de novo *Protein Interactants
G1 to S cell cycle control (*p *= 7.30 × 10^-3^)	■	■	■	■
G2_PATHWAY (*p *= 2.58 × 10^-3^)	▲	▲	■	■
				
Class I PI3K signaling events mediated by AKT (*p *= 0.14)	●	▼	▲	■
DNA damage response (*p *= 7.20 × 10^-3^)	●	●	▲	■
				
MAPK signaling pathway (*p *= 2.88 × 10^-2^)	●	►	▲	■
				
Glioma (*p *= 6.70 × 10^-3^)	►	►	▲	■
Non-small cell lung cancer (*p *= 5.80 × 10^-3^)	►	►	▲	■
Melanoma (*p *= 7.63 × 10^-3^)	►	►	▲	■
Endometrial Cancer (*p *= 5.59 × 10^-3^)	►	►	▲	■

Note: *p*-values are derived from one-tail Fisher's Exact test, before multiple test correction. Dividers and matching symbols indicate pathways groups within a consolidation method.

### p53 related pathway analysis

As a drill-down example, Table 3 shows consolidation results of seven pathways related to p53. Cisplatin is one of the most effective and widely used anticancer drugs. It is generally considered as a cytotoxic drug which kills cancer cells by damaging DNA and inhibiting DNA synthesis. On the other hand, the tumor suppressor protein p53 is considered as the "guardian of genome"[26]. It plays a critical role in eliciting cellular responses to cisplatin-induced DNA damage. To start the de novo consolidation, the *de novo *clustering methods based on gene membership, Gene Ontology, and protein-protein interaction were set for 500 clusters (membership500, GOSlim500 and PPI500). For *de novo *Membership, 3 pathway concepts were obtained (■, ▲, and ●) containing 2, 1 and 4 pathways respectively. Similarly, *de novo *Gene Ontology groups these pathways into 2 pathway concepts (■ and ●) with 3 and 4 pathways respectively. When we tried to group pathways by *de novo *PPI, all 7 pathways (■) listed in Table 3 were grouped together. As indicated by the different divisions, each method determines unique clusters, however, it is clear some of these p53 related pathways serve similar functions across different concept consolidation methods. Specifically, "Direct p53 effectors" and "p53 pathways" are always grouped together, with "p53 signaling pathway" possibly included in this group. By finding pathway concepts based on a variety of gene attributes, different functional connections are determined between the pathways.

### MMS case study

To demonstrate the capability of examining gene sets at pathway level, we also tested a second gene set that mimic the effect of cisplatin-induced DNA damage and inhibition of DNA synthesis. Ravi *et al. *[27] used a cell-based RNAi screen against the Drosophila genome and the alkylating agent methyl methanesulphonate (MMS). The screen determined a resultant set with 996 Drosophila genes required for DNA damage survival. Using this gene set they identified 13 pathways integral to DNA damage response. To compare their pathway analysis between two studies, we used Ensembl [28] to convert the Drosophila genes to 964 NCBI Entrez GeneIDs for the human orthologs.

The pathways found to be integral to DNA damage response included Base excision repair, Nucleotide Excision Repair, Mismatch Repair, Homologous Recombination Repair, RecQ helicases, DNA damage response, Proteasome, Glutathione Synthesis, TOR pathway, Basal Transcription, Ribosome, ATPase, and Notch signaling pathway. All of the pathways had equivalent human pathways except for RecQ helicases and ATPase. Using their resultant fly gene set converted to human orthologs we found enrichment scores for each, described in Table 4. Together with EC and WC, we performed *de novo *consolidation method with a cut-off of 100 clusters. In Table 4, we show each pathway's membership for clusters formed for each method. For *de novo *PPI, all pathways except Glutathione Synthesis were found in the same pathway concept (■). For *de novo *Membership, Notch and Tor pathways formed a separate concept (▲) and for *de novo *GOSlim, Base excision repair and Mismatch Repair formed a separate concept (●). EC separated everything into different clusters. WC combined Base excision repair, Mismatch Repair, Homologous Recombination Repair and DNA damage response (■) and separated every other pathway.

### HPD comparison

Chowbina *et al. *[14] determine 25 HPD pathways that contain some combination of the *BRCA1*, *FOXA1 *and *STK6 *genes. We downloaded their Pathway List and Pathway - Pathway Similarity Scores and by hand, found 7 pathway pairs with similarity score of 0.47 or higher among 9 of the original 25 pathways. Their similarity score is derived from pathway similarity based on pathway components (see reference for details). The pathways were Aurora A signaling (NCI-Nature), Signaling by Aurora kinases (NCI-Nature), Aurora B signaling (NCI-Nature), FOXA transcription factor networks (NCI-Nature), FOXA1 transcription factor network (NCI-Nature), FOXA2 and FOXA3 transcription factor networks (NCI-Nature), ATRBRCA_PATHWAY (Biocarta), and Fanconi Anemia pathway (Reactome). Indicated in Table 5 column 1, using HPD's similarity score, we grouped the Aurora, FOXA, ATRBRCA/Fanconi Anemia, and ATM/Repair of double-strand breaks pathways. Pathway Distiller finds 110 pathways that contain some combination of the *BRCA1*, *FOXA1 *and *STK6 *genes and 53 of them are enriched with a *p*-value less than 0.05. We tested if the different consolidation methods will cluster the 9 pathways like HPD. Described in Table 5, membership500 was the most similar to HPD's similarity group, exactly matching the FOXA and ATM/Repair of double-strand breaks pathways (●, ►). This similarity is to be expected because both HPD and Membership use gene set similarity as the scoring mechanism. Highlighted in the remaining columns of Table 5, Pathway Distiller's other methods, each utilizing different similarity measurements, find slightly different pathway groups.

We also extended our analysis of *TP53 *by compared HPD and Pathway Distiller's analysis of a single gene by searching on "p53" in HPD and inputting a file with "7157" in Pathway Distiller. Both yield all pathways that contain *TP53*. For HPD, we determined 20 pathways that group together into 6 clusters. By manual name comparison, we determined pathways similar to 9 of HPD's pathways. Described in Table 6, the 9 Pathway Distiller pathways map to four different HPD similarity groups (■, **▲**, ●, and ►). The cell cycle pathways were not grouped with any of the other pathways. The AKT, DNA repair and MAPK pathways were grouped. Finally all of the disease related pathways were grouped. Our *de novo *Membership, was most similar to HPD's groups and PPI the least similar, grouping all 9 pathways (■) together. Only the *de novo *methods were included in Table 6 because with a single gene resultant gene set, by definition, EC and WC will group all pathways.

## Discussion

Merging several pathway data sources allows for better coverage of existing information but is difficult because of the heterogeneous nature of the member pathways. A simple solution reduces the information for each pathway to its gene members. While this loses information that could be used to cluster pathways like directionality, we see that our consolidation methods achieve consolidation both *de novo *and with the experimentally focused data. The resulting pathway concepts are made up of pathways that have been found to share some functional characteristics. Using various methods for consolidation enables different views of the same data. For example, PPI and WC offer users consolidation based on interactions which could provide mechanisms for understanding how pathways are interconnected within a single concept. GOSlim on the other hand, focuses on functional connections according to GO Ontology annotations. Even within a single *de novo *method we provide various cut-offs that can find general to very specific measured similarities between pathways within a cluster. As each method has strengths, they also have weaknesses. The EC method is limited to only enriched pathways and will miss any non-enriched pathway. Because the other methods utilize a single measurement to assess similarity, they will sometimes miss connections that exist when the measurement does not fit the data.

For most research, EC and WC will provide the best overview of pathway concepts related to experiment specific enriched pathways and all pathways. Because EC is tightly aligned with enrichment results researchers interested in only connections between enriched pathways will typically rely on this method. However because WC includes non-enriched pathways, it is possible to find connections between enriched and non-enriched pathways.

Discussed further later, we saw that WC failed for Methionine Degradation I in our random testing even when the testing was based on interactions. This was due to the lack of interactions between the few gene members. However as we also saw in Table 2, this failure was mitigated by the successful concept development of the same pathway using EC. While we recognize that the goal of many pathway analysis tools is to find a single results and some confidence score, Pathway Distiller seeks to facilitate exploration of various results to aid a user in finding distinct functional connections between pathways in a pathway concept.

Pathway Distiller's supplied resultant gene set (cisplatin resultant gene set) is a good example of one of the challenges that our pathway enrichment consolidation addresses. The 177 pathways with *p*-values ≤ 0.05 are difficult to draw conclusions from. Shown in Table 1, the Enrichment Consolidation (EC) method reduces this to 12 pathway concepts, each represented by the most enriched among the pathways consolidated. This enables focused attention and readability. While the EC method only groups enriched pathways, the Weighted Consolidation (WC) method groups all pathways with at least one resultant gene. Out of the 2,462 human pathways, for example, there are 1,456 pathways with one of the cisplatin resultant genes that results in 318 pathway concepts. While this is too large compared with the EC method, it offers a different pathway description precision of the resultant gene set. Because it is not limited to enriched pathways, enriched and not enriched pathways are combined. Our web-based Pathway Distiller's result tables are column sortable and therefore we are able to find the lowest *p*-value for each pathway concept. In the case of cisplatin resultant gene set, there are 71 pathway concepts with at least one enriched pathway, much larger than 12 pathway concepts generated by EC method however small enough to browse for interesting information.

Often it is important to incorporate specific enrichment values within a single experimental design, for instance across time points in a single cell line treated with a compound. But among diverse experiments the most enriched pathway in one might have no relationship to another experiment. A *de novo *method for consolidating pathways independent of specific experiments that still handles the problems of set matching and numerical identification of subset similarity allows empirical comparison and consistent results. Like the WC method, both enriched and non-enriched pathways are included, however all gene members of each pathway were used to determine clusters, not only the resultant genes. Table 1 highlights the variation between the clustering methods and within the *de novo *methods with different cut-off values.

Measuring the number of interactions among a set of genes gives an estimation of their functional relatedness. The probabilities in the first column of Table 2 indicates that the interactions are unique among most pathway concepts and not due to random sampling of genes for the pathway concept found using the EC method. Clearly, in almost all of the cases, the pathway concepts are not combined without some relationship among the pathways as measured by interactions. The second column measures the randomness of the interactions in the matching clusters of the WC method. This method of validation fails for three of the pathway concepts we tested, two in EC and one in WC. This illustrates well the advantage of not using a "one size fits all" approach to consolidation. The two pathway concepts that fail for EC (Nucleotide Metabolism and Vitamin C in the Brain Pathway) both have few pathway gene members (6 and 4 respectively) and few interactions (1 and 0 respectively). Both matching clusters were significant for the WC method because each concept contains a slightly different set of member pathways. Interestingly, Methionine Degradation I (to homocysteine) fails in WC but was significant in EC again due to a slightly different set of member pathways. It is surprising that it fails in the interaction based validation when it was clustered due to weighted interactions until it is considered that there was only a single interaction among the genes in the concept, and this one interaction was very specialized to the pathway members of the concept.

Tables 3, 4, 5, 6 highlight the strengths of our consolidation methods. The tables should be read in a column-wise format; they include dividers to facilitate this. In other words, the divisions show how one clustering methods groups the pathway rows. Each division configuration represents a different way to cluster the rows of pathways.

From Table 3, one would expect grouping of pathways with a common relationship to p53. Gene Ontology divides the 7 pathways into two different clusters and the two different clusters have significance in terms of normal cells and those dealing with DNA damage response. Protein Interactions and the EC method group all pathways together. Gene Membership and WC method group the 7 pathways into three and four pathway concepts respectively. The WC method follows the grouping of the Gene Membership except it separates the p53-dependent and p53-independent pathways, clearly offering different precision of pathway representation.

Not only does pathway consolidation make the data more manageable, readable and publishable, it could focus attention to pathways not previously considered. For example, Ravi *et al. *[27] determined 13 pathways related to DNA damage response, 11 corresponding human pathways are shown in Table 4. Similar to their findings, when we utilized the human ortholog resultant gene set for their RNAi screen, not all of the pathways were enriched, therefore simple enrichment methods would fail to draw attention to one of these pathways. Our different but complementary consolidation methods can group both enriched and non-enriched pathways together. For example, Gene membership100 finds Base excision repair, Nucleotide Excision Repair, Mismatch Repair, Homologous Recombination Repair (HRR) and DNA damage response all together in a single concept (■). Only Nucleotide Excision Repair would have been apparent in the initial pathway enrichment step with a *p*-value of 1.99 × 10^-4^. But, by looking at pathways also clustered with Nucleotide Excision Repair in the Consolidation Results grid, one might find connections between enriched and non-enriched pathways. Ravi *et al. *created a similar hypothesis of connections between these pathways by hand. In this example, all pathways except for Glutathione Metabolism are contained in the same concept in at least one method and some are in the same concept for all methods. Because Glutathione Metabolism is never clustered with the other pathways this offers a chance for exploration of the differences between Glutathione Metabolism and the other pathways. Similarly, the possible functional connections between Notch and Tor pathways (membership100) and Base Excise Repair and Mismatch repair (PPI100) might lead to novel avenues for research.

Pathway Distiller found 53 and 105 enriched pathways when processing the MMS and TP53 case studies, respectively. Functional connections in the form of interactions, gene membership and GO Ontology between pathways will enable users to focus attention on meaningful groups instead of many individual members.

**Figure 4:**
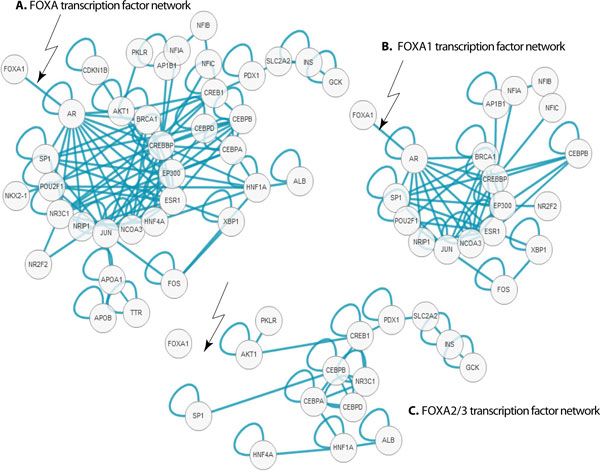
**Interaction network for FOXA, FOXA1 and FOXA2/3 transcription factor networks**. FOXA1 and AR interaction is noted (present/missing) to highlight if AR is in pathway or not.

Table 5 shows that the FOXA, FOXA1 and FOXA2/FOXA3 pathways were grouped by HPD. Figure 4 illustrates the overlapping nature of the FOXA transcription factor network and the FOXA1 transcription factor network. HPD and Pathway Distiller's membership500 combined the three pathways into a single cluster, however PPI500 and WC did not. As indicated in the figure there is some amount of overlap between the three pathways. The FOXA1 gene is connected to the network through a single gene, AR. AR is not included in the FOXA2/FOXA3 transcription factor network. Because HPD and membership500 rely on overlap of gene sets, it is not surprising that the cluster contains all three pathways. Conversely, the interaction-based methods split the pathways into different concepts and in this case because of the functional importance of AR.

In the second comparison with HPD, their similarity scores placed MAPK signaling pathway and the cancer pathways into different clusters. MAPK signaling pathway was included in the cancer related cluster for Pathway Distiller's membership500 cluster. Pathway Distiller also includes Bladder, Colorectal cancer, Pancreatic cancer, Chronic myeloid leukemia, Thyroid, Prostate, and small cell lung cancers. Dhillion *et al. *[29] describe how MAPK features prominently in cancer. In retrospect, the membership500 cluster could have hinted at the same conclusion.

## Conclusions

Pathway enrichment is an important tool when making sense of gene expression data, however the information must be in a form conducive to examination and evaluation. Each of the publically available pathway data sources has strengths and weaknesses. In order to exploit the information, the combination of these data sources is essential, but only if we can avoid overlapping and maintain the independence between the pathway concepts. The proposed pathway consolidation is important to make the combined data manageable, but still accurately describing the original data. Three methods were discussed in the paper: one focusing on pathways determined enriched in a given experiment, another looks at both enriched and non-enriched pathways, but using only the experiment's resultant genes, and the final grouping the pathways in a *de novo*, experiment independent manner. In addition, the web-based Pathway Distiller implementation (http://cbbiweb.uthscsa.edu/PathwayDistiller/) will enable access to a variety of pathway data sources, pathway enrichment given resultant gene sets and our diverse consolidation methods. The default option consolidation method, Enrichment Consolidation and Weighted Consolidation, are the most descriptive representation of the enrichment results, but the other methods offer additional views of the results focusing on gene annotation information and the overlap of enriched and non-enriched pathways within concepts.

Pathway Distiller will benefit a scientist who needs a simple-to-use yet sophisticated pathway consolidation and enrichment method to find functional insight for their genotypic data. It allows them to use four steps to retrieve concept grouping and then the additional ability to further explore concept relationships if desired.

## Competing interests

The authors declare that they have no competing interests.

## Authors' contributions

MSD developed the Pathway Distiller framework, participated in the design and evaluation of the methods and drafted the manuscript. ZA developed initial version of one method. US performed the statistical analysis. RD participated in the design of the methods. AJRB participated in the design and evaluation of the methods, participate in the design of the Pathway Distiller framework and helped draft the manuscript. YC participated in the design and evaluation of the methods, participate in the design of the Pathway Distiller framework and helped draft the manuscript. All authors read and approved the final manuscript.
